# Brain activation patterns associated with cue reactivity and craving in abstinent problem gamblers, heavy smokers and healthy controls: an fMRI study

**DOI:** 10.1111/j.1369-1600.2010.00242.x

**Published:** 2010-10

**Authors:** Anna E Goudriaan, Michiel B de Ruiter, Wim van den Brink, Jaap Oosterlaan, Dick J Veltman

**Affiliations:** 1 Department of Psychiatry, Academic Medical Center, University of AmsterdamThe Netherlands; 2 Amsterdam Institute for Addiction ResearchAmsterdam, The Netherlands; 3 Department of Psychiatry, VU University Medical CenterAmsterdam, The Netherlands; 4 Department of Clinical Neuropsychology, VU University AmsterdamAmsterdam, The Netherlands

**Keywords:** Addiction, cue reactivity, fMRI, impulse control disorder, nicotine dependence, pathological gambling

## Abstract

Abnormal cue reactivity is a central characteristic of addiction, associated with increased activity in motivation, attention and memory related brain circuits. In this neuroimaging study, cue reactivity in problem gamblers (PRG) was compared with cue reactivity in heavy smokers (HSM) and healthy controls (HC). A functional magnetic resonance imaging event-related cue reactivity paradigm, consisting of gambling, smoking-related and neutral pictures, was employed in 17 treatment-seeking non-smoking PRG, 18 non-gambling HSM, and 17 non-gambling and non-smoking HC. Watching gambling pictures (relative to neutral pictures) was associated with higher brain activation in occipitotemporal areas, posterior cingulate cortex, parahippocampal gyrus and amygdala in PRG compared with HC and HSM. Subjective craving in PRG correlated positively with brain activation in left ventrolateral prefrontal cortex and left insula. When comparing the HSM group with the two other groups, no significant differences in brain activity induced by smoking cues were found. In a stratified analysis, the HSM subgroup with higher Fagerström Test for Nicotine Dependence scores (FTND M = 5.4) showed higher brain activation in ventromedial prefrontal cortex, rostral anterior cingulate cortex, insula and middle/superior temporal gyrus while watching smoking-related pictures (relative to neutral pictures) than the HSM subgroup with lower FTND scores (FTND M = 2.9) and than non-smoking HC. Nicotine craving correlated with activation in left prefrontal and left amygdala when viewing smoking-related pictures in HSM. Increased regional responsiveness to gambling pictures in brain regions linked to motivation and visual processing is present in PRG, similar to neural mechanisms underlying cue reactivity in substance dependence. Increased brain activation in related fronto-limbic brain areas was present in HSM with higher FTND scores compared with HSM with lower FTND scores.

## INTRODUCTION

Pathological gambling (PG) is a fairly common disorder with an estimated point prevalence of approximately 1% (Welte *et al*. 2001). PG often results in severe psychosocial problems (Petry & Kiluk 2002; Potenza *et al*. 2002). Currently, PG is classified as an impulse control disorder, but the diagnostic criteria closely resemble those of substance dependence. In addition, recent studies have shown neurobiological similarities between PG and substance dependence (Petry & Kiluk 2002; Potenza *et al*. 2002; Goudriaan *et al*. 2004). As a consequence, some authors have proposed to reclassify PG as a behavioural addiction in DSM-V (Petry 2006; Potenza 2006).

Increased cue reactivity coupled with heightened attention for addiction-related cues represents an important mechanism in the development of addictive behaviors (Goldstein & Volkow 2002) and may promote relapse in substance dependence (Cooney *et al*. 1997; Kosten *et al*. 2006; Marissen *et al*. 2006). Functional imaging studies using cue-exposure paradigms in nicotine, alcohol and cocaine dependence have reported increased ventral prefrontal, insular, amygdala, striatal, and thalamic activity, brain regions associated with emotion processing and motivational behavior. In addition, attentional and cognitive control circuitry has been implicated in neuroimaging cue reactivity studies, indicated by increased dorsolateral prefrontal, anterior cingulate cortex and parietal activation (Kilts *et al*. 2001; Tapert *et al*. 2004; David *et al*. 2005; Kosten *et al*. 2006; McBride *et al*. 2006; Franklin *et al*. 2007).

About 50% of pathological gamblers who try to quit experience a relapse with seriously negative consequences (Hodgins & el Guebaly 2004), and other studies indicate frequent relapses in treatment-seeking pathological gamblers (Ledgerwood & Petry 2006). Because cue reactivity is a key mechanism in the development of addictive disorders, and because it has been associated with a higher risk of relapse in substance dependence (Cooney *et al*. 1997; Kosten *et al*. 2006; Marissen *et al*. 2006), investigating the neurobiological mechanisms of cue reactivity in this population is highly relevant. So far, only two functional magnetic resonance imaging (fMRI) studies on exposure to gambling-related cues in pathological gamblers have been published (Potenza *et al*. 2003; Crockford *et al*. 2005). Both studies employed video fragments of gambling-related and various control scenes, but yielded inconsistent results. In the first study among 10 pathological gamblers and 11 normal controls, PG subjects revealed decreased, rather than increased activation in the ventral anterior cingulate cortex, orbitofrontal cortex, basal ganglia and thalamus during gambling-associated versus control epochs. Increased activation during viewing of gambling-related material was found in the occipital lobe only (Potenza *et al*. 2003). In the second study in 10 pathological gamblers and 10 healthy controls (HC) (Crockford *et al*. 2005), PG subjects showed higher brain activation in response to gambling stimuli in the left occipital cortex, left fusiform gyrus, right parahippocampal gyrus and right prefrontal areas, compared with HC.

Thus, whereas these PG studies indicate increased activation of brain regions involved in attention, memory and visual processing, no evidence for abnormally increased activity in limbic structures during processing of gambling cues was found (e.g. increased activation in amygdala), unlike neuroimaging studies on cue reactivity in substance dependence (Kilts *et al*. 2004; Tapert *et al*. 2004; Kosten *et al*. 2006; McBride *et al*. 2006; Franklin *et al*. 2007). Possible reasons for this discrepancy are the use of videos instead of pictures and lack of power because of small sample sizes. Furthermore, both studies enrolled gamblers recruited through advertisements, and neither study investigated whether treatment-seeking problem gamblers (PRGs) would differ in cue reactivity to gambling cues from normal controls. In an fMRI study focusing on the processing of rewards in pathological gamblers (Reuter *et al*. 2005), a blunted response to wins versus losses was found in the limbic reward areas in pathological gamblers versus HC. When presenting pathological gamblers with gambling videos, the limbic system may thus be relatively underactivated because of a diminished response to gambling situations in which money is gained. Given this blunted response to monetary gains, the investigation of limbic activation to gambling cues versus neutral cues that do not include monetary gain may provide insight in cue reactivity to general gambling cues.

In the present study, we wanted to address these issues by investigating brain activation patterns to gambling or smoking cues in chronic PRGs seeking treatment, heavy smokers (HSM) and non-smoking non-gambling healthy controls (HC). We employed an event-related picture paradigm (George *et al*. 2001; Myrick *et al*. 2004; Smolka *et al*. 2006) because this provides optimal flexibility with regard to stimulus timing and avoids modeling problems which may arise when analyzing video paradigm fMRI data. In order to compare cue reactivity in PRG to cue reactivity of a substance-dependent group, a comparison group of HSM was included as well. An HSM control group was chosen because the neurotoxic effects of nicotine are limited compared with those of other drugs of abuse, such as alcohol (Sullivan 2003; Mudo, Belluardo & Fuxe 2007). Based on previous cue-reactivity studies in substance dependence, we hypothesized that gambling cues in PRG and smoking cues in HSM would elicit higher brain reactivity compared with brain reactivity in healthy non-smoking controls in brain regions associated with emotion processing and motivational behavior such as the amygdala, ventral striatum and ventral prefrontal cortex, and in attention and cognitive control-related brain areas such as the dorsal prefrontal cortex and anterior cingulate cortex (ACC). In addition, the relation between cue-related brain activity and subjective craving in PRG and HSM was studied. We hypothesized that subjective craving would be associated with increased activation in emotion and motivation-related brain areas in PRG and HSM.

## MATERIALS AND METHODS

### Subjects

Nineteen treatment-seeking PRG (four left-handed), 19 HSM (three left-handed) and 19 non-smoking HC (one left-handed), all males, participated in this study. For two PRG, one HSM and two HC, magnetic resonance imagine (MRI) data could not be (completely) acquired because of scanner failures. Therefore, 17 PRG, 18 HSM and 17 HC constituted the three groups used for statistical analysis. The PRG were recruited from two Dutch addiction treatment centers. The HSM and the HC group were recruited through advertisements in newspapers.

The main inclusion criterion for PRG was current treatment for gambling problems. PRG were interviewed with section T of the Diagnostic Interview Schedule (Robins *et al*. 1998) to assess the diagnostic criteria for a DSM-IV-TR diagnosis of PG. In addition, the South Oaks Gambling Screen (SOGS; Lesieur & Blume 1987) was administered as a measure of problem gambling severity. Two PRG failed to meet the criteria of a current DSM-IV-TR PG diagnosis. However, because they did meet two PG criteria currently, met PG criteria in the past and their SOGS scores (7 and 8, respectively) were similar to the PRG that did fulfill diagnostic criteria for PG (see Table 1; mean SOGS score = 9.6 ± 2.6), these PRG were included in the analyses. All PRG were abstinent from gambling for at least 1 week. HSM were included if they smoked at least 15 cigarettes per day, and did not engage in gambling activities more than twice a year. HSM were current smokers who engaged in an experimental smoking cessation as part of this study. The Fagerström Test for Nicotine Dependence (FTND) served as an indicator of nicotine dependence severity (Heatherton *et al*. 1991). No minimum score on the FTND was required for HSM. HSM had to be overnight smoking abstinent, filled out questionnaires in the morning and were scanned in the afternoon (16–18 hours abstinent). Abstinence was confirmed with a breath carbon monoxide measurement in the morning, using a micro+ Smokerlyzer (Bedfont Scientific, Ltd., Rochester, UK). HC never smoked, did not have a history of problem gambling and did not engage in gambling activities more than twice in the last year.

**Table 1:** Demographic characteristics for problem gamblers, heavy smokers and healthy controls

	*Problem gamblers (n* = *17)*	*Heavy smokers (n* = *18)*	*Healthy controls (n* = *17)*	*ANOVA, F test and significance (P value, 2-tailed)*
Age, mean (SD)	35.3 (9.4)	33.8 (9.1)	34.7 (9.7)	*F*(2,49) = 0.11, *P* = 0.89
Education level, mean (SD)	4.1 (0.9)	4.1 (1.1)	4.3 (1.2)	*F*(2,49) = 0.15, *P* = 0.86
Pathological Gambling Lifetime Diag^a^ No. (%)	14 (82%)	0	0	
Pathological Gambling 12 M Diag^a^, No. (%)	11 (65%)	0	0	
South Oaks Gambling Screen, 12 months, scale range 0–14; mean (SD)	9.6 (2.6)	0 (0)	0 (0)	
Gambling-related debts in € (SD)	58.843,− (67.131,−)	–	–	
Fagerström Test for Nicotine Dependence, mean (SD)	2.0 (−)^b^	4.0 (1.5)	0 (0)	
Mean *n* cigarettes/day (SD)	<5 (−)^b^	17.2 (3.8)	0 (0)	
Breath carbon monoxide (BCO) levels in particles per million (SE)	2.2 (0.9)	9.0 (0.9)	2.0 (0.9)	*F*(2,51) = 21.24, *P* < 0.001
CIDI Anxiety 12 M Diag, No. (%)	3 (17%)	0	0	
CIDI Depression 12 M Diag, No. (%)	3 (17%)	0	0	
Total comorbidity, No. (%)	4 (22%)	0	0	
Conners Adult ADHD Rating Scale, mean (SD)	52.5 (14.2)	46.1 (11.6)	44.5 (6.1)	*F*(2,49) = 2.42, *P =* 0.099
Beck Depression Inventory, mean (SD)	11.1 (12.0)	4.4 (4.2)	3.8 (4.1)	*F*(2,49) = 4.7, *P =* 0.013
Alcohol Use Disorders Identification Test—C	4.5 (2.7)	4.8 (2.6)	3.4 (2.1)	*F*(2,49) = 1.7, *P* = 0.19
Gambling Urge Before Cue Reactivity Test	1.43 (.75)	1.18 (.33)	1.01 (.07)	See text
Gambling Urge After Cue Reactivity Test	1.76 (.70)	1.19 (.32)	1.02 (.10)	See text
Smoking Urge Before Cue Reactivity Test	1.28 (.66)	3.91 (1.27)	1.01 (.05)	See text
Smoking Urge After Cue Reactivity Test	1.37 (.86)	4.11 (1.36)	1.00 (.00)	See text

a Pathological gambling was diagnosed with Diagnostic Interview Schedule Section T.

b One gambler smoked less than five cigarettes a day. LT Diag = Lifetime Diagnosis; 12 M Diag = 12-Month Diagnosis; CIDI = Composite International Diagnostic Interview.

Exclusion criteria for all groups were: age under 18 years; difficulty reading Dutch; use of psychotropic medication; a lifetime diagnosis of schizophrenia or psychotic episodes; a 12-month diagnosis of manic disorder, assessed with the respective sections of the Composite International Diagnostic Interview (CIDI; Heatherton *et al*. 1991; World Health Organization 1997); current treatment for mental disorders other than those under study; physical conditions known to influence cognition or motor performance (e.g. multiple sclerosis, rheumatic disease); positive urine screen for alcohol, amphetamines, benzodiazepines, opioids or cocaine; consumption of more than 21 units of alcohol per week. Groups were mutually exclusive with regard to the psychiatric disorder under study. For instance, PRG and HC did not smoke (with the exception of one PRG who smoked less than five cigarettes a day). Additional exclusion criteria for HC and HSM, but not for PRG, were presence of anxiety disorders (CIDI-section D), depression (CIDI-section E), obsessive–compulsive disorder (CIDI-section K), post-traumatic stress disorder (CIDI-section K) and attention-deficit/hyperactivity disorder (Conners ADHD Rating Scales; Conners & Sparrow 1999). PRG with these comorbid disorders were not excluded, because problem gambling is highly comorbid with these disorders. The severity of depression symptoms was assessed with the Beck Depression Inventory (BDI-II; Beck *et al*. 1996). Problematic alcohol use was screened with the Alcohol Use Disorders Identification Test-Consumption (Bush *et al*. 1998).

In addition to the Cue Reactivity Task, a probabilistic reversal learning task, a planning task and a stop signal task were administered. Results from the reversal learning task and the planning task are reported elsewhere (de Ruiter *et al*. 2009). The ethical review board of the Academic Medical Center approved the study and written informed consent was obtained. Participants were reimbursed with €50 transferred to their bank account following participation.

### fMRI paradigm: Cue Reactivity Task

**Figure 1:**
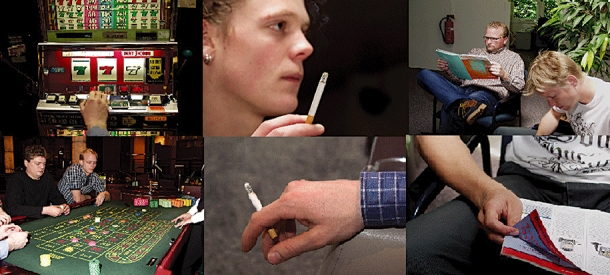
Examples of gambling stimuli (left), smoking-related stimuli (middle) and neutral stimuli (right)

A picture two-choice response task was used (for examples of pictures, see Fig. 1). Pictures were matched for complexity as follows: an equal number of overview pictures and detail pictures was selected for each condition (e.g. several persons gambling, smoking or talking, versus detailed pictures of a hand at a slot machine, a hand with a cigarette, a hand with a magazine). Second, to match for picture complexity and comparability, all pictures were taken in a similar naturalistic setting (e.g. all pictures with multiple persons were taken with multiple objects in the background), only males were included on pictures, and care was taken to match for emotional expressions between the different pictures, by including only photos with neutral face expressions. Thirty gambling pictures, 30 smoking-related pictures, 30 neutral pictures and 30 low-level baseline pictures were presented randomly, with the restriction that a stimulus of the same stimulus category was not presented more than three times in a row. Low-level baseline pictures with arrows pointing to the left or right were presented, and a left or right response had to be given, in order to be able to compare complex picture processing compared with low-level visual processing. In the gambling, smoking-related and neutral pictures, participants had to press a response button with their left index finger when a face was present in the picture and had to press a response button with their right index finger when no face was present. Fifty percent of all pictures within each category contained a face. Each picture was presented for a fixed period of 5 seconds, and participants were requested to respond within this time period. When no response was made after 5 seconds, the task proceeded. A 2.5-second blank screen was presented between each picture. No feedback was given about right or wrong responses. The scanning session lasted 15 minutes; each of the gambling, smoking-related and neutral pictures was presented once. Subjects were not encouraged to respond as quickly as possible. The task was explained and practiced outside the scanner using other pictures. The performance parameter for the task was mean reaction time to the pictures in each stimulus category.

### Urge questionnaires

An 8-item gambling urge questionnaire, range 1–7 (MN Potenza & SS O'Malley, unpublished data) and a 10-item smoking urge questionnaire, range 1–7 (Tiffany & Drobes 1991), were included to assess levels of gambling and nicotine craving, respectively. Participants filled out the urge questionnaires before and immediately after fMRI scanning.

### Imaging acquisition and preprocessing

Imaging data was obtained using a 3.0 Tesla Philips Intera full-body fMRI scanner equipped with a standard SENSE RF head coil (Quasar gradient system, Philips Medical Systems BV, Eindhoven, the Netherlands) located at the Academic Medical Center, Amsterdam. While participants performed the task, T2*-weighted echo planar images, sensitive to blood oxygenation level-dependent (BOLD) contrast were acquired (35 axial slices, voxel size 3 × 3 × 3 mm, interslice gap 0.3 mm, matrix size 64 × 64 mm, bandwidth 90 kHz, TE 35 ms, repetition time 2.28 seconds), covering the entire brain except for the inferior regions of the cerebellum. A sagittal T1-weighed structural scan (voxel size 1 × 1 × 1 mm, 170 slices) was made in order to co-register it with the fMRI data. Imaging analysis was done using SPM2 (Statistical Parametric Mapping; Wellcome Department of Cognitive Neurology, London, UK). Images were slice-timed, reoriented and realigned to the first volume. Next, T1-coregistered volumes were normalized to an SPM T1- template (using 12 linear parameters and a set of non-linear cosine basis functions), and spatial smoothing was performed using an 8 mm FWHM Gaussian kernel.

### Statistical analysis

Group differences in demographic and clinical data were analyzed using univariate analysis of variance (ANOVA) and Tukey's *post hoc* tests. Group differences in educational level were analyzed using Pearson's chi-square test. ANOVAs were used to analyze performance data (mean reaction time) with group as between-subject factor (PRG, HSM and HC), and stimulus category (gambling versus neutral, smoking-related versus neutral, or low-level baseline versus neutral) as within-subject factor, using group contrasts. ANOVA was used to analyze urge ratings (mean gambling urge, mean smoking urge), with time (before and after task completion) as within-subject factor. All analyses were performed two-tailed.

The mean FTND score in the HSM group was low (M = 4.0; SD = 1.5) compared with FTND scores in smokers reported in other fMRI cue reactivity studies (Franklin *et al*. 2007, FTND = 4.8; McClernon *et al*. 2007, FTND = 6.4; McClernon, Kozink & Rose 2008, FTND = 6.5), and no nicotine dependence diagnoses were available for the HSM, as in other studies (Brody *et al*. 2002). Therefore, exploratory analyses were done, comparing HSM with high FTND scores (*n* = 10, FTND-high group M = 5.4, SD = 0.5) to HSM with low FTND scores (*n* = 8, FTND-low group: M = 2.9, SD = 1.0), after a median split was made. In the PRG group, no split was made between high or low severity PRG, because severity of gambling problems in our sample, as assessed with the SOGS, was comparable with severity reported in other studies in treatment-seeking pathological gamblers.

The fMRI data were analyzed in the context of the general linear model, using delta functions convolved with a synthetic hemodynamic response function to model responses to each stimulus type. For each comparison of interest, single-subject contrast images were entered into second-level (random effects) analyses. To investigate differential processing of addiction relevant stimuli between groups, one-way ANOVAs were performed and interaction effects were computed for gambling versus neutral pictures in the PRG versus the HC or HSM, and for smoking-related versus neutral pictures in the HSM (HSM total group; FTND-high group; FTND-low group) versus the PRG or the HC. Main effects and interaction effects were analyzed with one-way ANOVA implemented in SPM2 and are reported with a cluster size restriction of 10 voxels at *P* < 0.05 corrected for multiple comparisons according to the Family Wise Error method (Tiffany & Drobes 1991; Nichols & Hayasaka 2003). Group interactions are reported with a cluster size restriction of 5 voxels at *P* < 0.001, masked with the appropriate main effect.

Gambling or smoking-related pictures versus neutral pictures were chosen for our main group-interaction contrast, because this contrast is most specific for the cue-reactivity effect: reactivity to addiction specific cues versus cues not related to addiction. The comparison of addiction-related pictures versus baseline would include various non-specific visual processes (such as stimulus processing, object recognition) that are activated when watching visually complex stimuli compared with very simple visual stimuli (an arrow pointing to the left or right). An interaction between addiction-related pictures and baseline would therefore be less specific, because visual processing would then interact with cue reactivity effects. However, in addicted populations, it is important to establish that baseline visual interpretation is similar in both addicted persons and non-addicted groups. In another study from our group, it was found that addicted persons had a larger brain response to neutral pictures compared with baseline (Zijlstra *et al*. 2009). Therefore, we also present the contrast neutral versus baseline, to demonstrate that neutral pictures generated similar activation patterns across groups.

In addition, the potential influence of left-handedness on brain activity patterns was investigated by performing all analyses with and without left-handed participants. The activity patterns found after excluding left-handed participants were very similar to those obtained when including both left- and right-handed participants. Therefore, in the Results section, we only present data based on the whole sample.

Regression analyses were performed for the PRG and HSM separately, to investigate whether brain activation in response to addiction-related stimuli (gambling and smoking stimuli, respectively) *versus* the neutral pictures correlated with self-reported craving after scanning. Regression analyses were also conducted to investigate whether co-morbid ADHD [Conners Adult ADHD Rating Scales (CAARS) scores] and depressive symptoms (BDI-II scores) correlated with cue-reactivity-related brain activation (addiction-related pictures versus neutral pictures). Because the PRG scored somewhat higher on the CAARS, and much higher on the BDI-II than the other two groups (see Table 1), these analyses were done separately for each group. Four PRG had co-morbid psychiatric disorders (anxiety and/or depression). Therefore, group interactions including PRG were analyzed both with and without these co-morbid participants.

## RESULTS

### Demographic and clinical results

Table 1 summarizes demographic and clinical characteristics for the three groups. PRG had an average of almost €60 000 in gambling-related debts. Breath carbon monoxide levels were higher for the HSM, compared with PRG and HC. PRG obtained higher scores on the CAARS and BDI-II than both HSM and HC.

### Results for performance data and craving ratings

Mean reaction times to gambling pictures (M: 1143 ms, SD: 340) were longer than mean reaction times to neutral pictures (M: 1006 ms, SD: 311), *F*(1,49) = 50.1, *P* < 0.0001; mean reaction times to smoking pictures (M: 929 ms, SD: 235) were shorter than mean reaction times to neutral stimuli (*F*(1,49) = 12.9, *P* < 0.0001; and mean reaction times to the low level baseline condition (M: 717 ms, SD: 169) were shorter than to the neutral stimuli, *F*(1,49) = 80.3, *P* < 0.0001, but no stimulus type by group interactions were present (all group by stimulus contrasts *F* values < 1, NS). Accuracy was high; mean number of errors summed across all conditions was 1.2, and no differences in number of errors between groups or conditions were found (*F* < 1, NS). ANOVA indicated that craving for smoking before scanning was higher in the HSM compared with HC, *F*(1,34) = 87.4, *P* < 0.0001, and compared with PRG *F*(1,34) = 57.8, *P* < 0.0001. Craving did not differ between the FTND-high group and the FTND-low group, *F*(1,17) < 1, NS. No difference between smoking craving before and after the cue reactivity task in the total group of HSM *F*(1,17) = 1.42, *P* = 0.25, nor in the FTND-high group versus the FTND-low group, *F*(1,16) = .29, *P* = 0.60 was present. Craving for gambling was higher in PRG compared with HSM and HC, *F*(2,51) = 6.92, *P* < 0.002, and a trend for increased gambling craving after the cue reactivity task was observed in PRG, *F*(1,16) = 3.18, *P* = 0.09, partial η^2^ = 0.17 (defined as a large effect size, Stevens 1996).

### fMRI cue reactivity

#### Main effects (pictures versus baseline)

**Figure 2:**
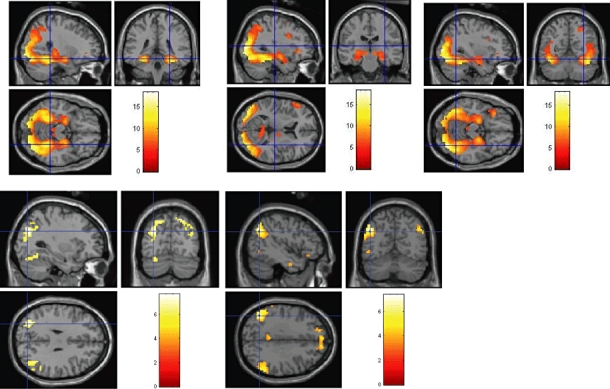
Activation patterns across groups for neutral pictures versus low-level baseline pictures (top left panel), gambling pictures versus low-level baseline pictures (top middle panel), smoking pictures versus low-level baseline pictures (top right panel), gambling pictures versus neutral pictures (lower left panel), smoking-related pictures versus neutral pictures (lower right panel)

The main effects of viewing neutral pictures versus low-level baseline pictures were observed in all three groups mainly in the ventral visual stream (occipital lobe: middle, inferior and lingual gyrus), as well as in areas related to reward/motivation, and attention and cognitive control; medial temporal lobe including the amygdala, bilateral dorsolateral prefrontal cortex (DLPFC), as well as bilateral posterior thalamus, see Fig. 2, left panel. For gambling versus baseline pictures and smoking-related versus baseline pictures, similar regions were identified. In addition, we found bilateral activation of the ventrolateral prefrontal cortex (VLPFC) for gambling and smoking-related pictures versus baseline pictures, as well as dorsomedial prefrontal cortex activation for gambling pictures versus baseline pictures (Fig. 2, middle and right panels, respectively).

#### Group interactions

**Figure 3:**
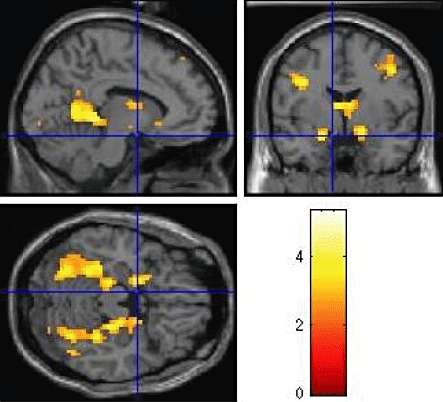
Group interaction: Areas highlighted for higher activation in problem gamblers (PRG) versus the pooled sample of healthy controls (HC) and heavy smokers (HSM) at coordinates −9, 0, −18. Exclusion of PRG with co-morbid psychiatric disorders resulted in similar results, although differences in dorsolateral prefrontal cortex (DLPFC) in PRG compared with HC, and in right amygdala activation and left DLPFC in PRG compared with HSM ceased to be statistically significant. No significant relations between Beck Depression Inventory-II, or Conners Adult ADHD Rating Scales scores and blood oxygenation level-dependent response when viewing gambling pictures versus neutral pictures were present in PRG, HSM or HC

For neutral pictures versus low-level baseline pictures, no significant group interaction effects were observed. For gambling pictures versus neutral pictures, we found greater activation in left occipital cortex, bilateral parahippocampal gyrus, right amygdala and right DLPFC in PRG relative to HC. Relative to the HSM, PRG showed higher bilateral occipital cortex, bilateral parahippocampal gyrus, bilateral amygdala, bilateral DLPFC and left VLPFC activation when viewing gambling pictures versus neutral pictures (Table 2 and Fig. 3). Similar group differences were observed when PRG with co-morbid psychopathology were excluded, although differences in DLPFC activation in PRG compared with HC, and differences in activation in right amygdala and left DLPFC in PRG compared with HSM ceased to be statistically significant.

**Table 2:** Cue Reactivity Task: BOLD activations for main effects (neutral/gambling/smoking-related pictures versus low-level baseline pictures); group interactions (gambling pictures versus neutral pictures, and smoking-related pictures versus neutral pictures); correlations between BOLD activations and self-reported craving

	*Neutral versus baseline*					*Gambling versus baseline*					*Smoking-related versus baseline*					*Gambling versus neutral*					*Smoking-related versus neutral*				
		*MNI coordinates*					*MNI coordinates*					*MNI coordinates*					*MNI coordinates*					*MNI coordinates*			
*Main effects*	*L/R*	*x*	*y*	*z*	*Z value*	*L/R*	*x*	*y*	*z*	*Z value*	*L/R*	*x*	*y*	*z*	*Z value*	*L/R*	*x*	*y*	*z*	*Z value*	*L/R*	*x*	*y*	*z*	*Z value*
Prefrontal																									
Dorsolateral																									
		−42	9	33	7.43	L	−42	12	33	7.44											L	−3	54	36	5.17
		42	15	30	7.42	R	54	24	30	7.77	R	42	15	33	6.92	L	−9	27	57	5.99					
Ventrolateral	L	−30	33	−9	5.78		−33	33	12	6.59											L	−33	48	6	3.87
	R	36	36	−6	6.32		36	33	−6	7.52	R	36	33	−6	5.59										
Medial																					R	3	54	0	4.75
Temporal																									
Middle temporal		−33	−18	−21	6.43	L	−30	−21	−21	6.53	L	−30	−15	−15	6.77						L	−39	6	−15	4.83
	R	33	−15	−15	7.29	R	30	−21	−21	7.22	R	33	−15	−15	6.58										
Medial temporal																									
Parahippocampal gyrus	L	−27	−33	−21	12.01	L	−33	−48	−18	7.90	L	−24	−33	−18	7.80	L	−27	−45	−15	6.07					
	R	27	−48	−15	14.30	R	33	−48	−18	11.00	R	36	−48	−24	13.25	R	33	−45	−12	7.06					
Amygdala region						L	−21	−6	−18	8.60	L	−21	−6	−15	7.83										
						R	24	−6	−18	9.76	R	21	−6	−15	7.81										
Occipital lobe																									
Occipital gyrus		−27	−84	−9	15.35		−24	−84	−6	12.23		−30	−84	−9	14.24	L	−36	−66	27	5.48	L	−45	−63	33	5.86
		−39	−78	9	13.68		−33	−72	21	8.90		−36	−81	12	13.65	R	45	−63	30	5.49	R	45	−63	39	4.80
Fusiform gyrus	R	36	−45	−15	10.39	R	39	−63	−15	10.28	R	39	−63	−15	11.15	R	60	−54	12	5.86	R	60	−36	−3	5.12
Occipital gyrus		27	−84	−9	11.50		24	−84	−15	15.03		24	−84	−15	13.65										
		24	−81	12	8.41		24	−90	12	12.85		24	−90	12	12.65										
Posterior thalamus	L	−18	−30	−3	11.58	L	−21	−30	−3	10.21	L	−21	−30	−3	11.27										
	R	18	−30	−3	9.43	R	21	−30	−3	10.94	R	21	−30	−3	6.92										

*Group Interactionsa*																									
	
*Gambling vs. Neutral Pictures*	*Problem Gamblers > Healthy Controls*					*Problem Gamblers > Heavy Smokers*																			
		*MNI coordinates*					*MNI coordinates*																		
	*L/R*	*x*	*y*	*z*	*Z value*	*L/R*	*x*	*y*	*z*	*Z value*															
	
Prefrontal						L	−48	9	33	3.64															
Dorsolateral	R	39	7	30	3.22	R	45	0	42	4.31															
Ventrolateral						L	−42	24	3	3.30															
Medial Temporal																									
Parahippocampal Gyrus	L	−18	−24	−21	3.43	L	−27	−45	−15	4.04															
	R	21	−27	−21	3.52	R	27	−36	−21	4.01															
						R	30	−60	−15	3.45															
Amygdala						L	−21	9	−18	3.54															
	R	15	0	−15	3.34	R	15	5	−10	3.43															
Occipital/Temporal junction						R	57	−60	3	3.56															
Middle Occipital Gyrus	L	−33	−78	12	3.40	L	−33	−78	12	4.89															
		−39	−72	12		R	27	−72	33	3.20															

a Group interaction are reported at *P* < 0.001 unless indicated otherwise.

BOLD = blood oxygenation level-dependent; MNI = Montreal Neurological Institute.

**Figure 4:**
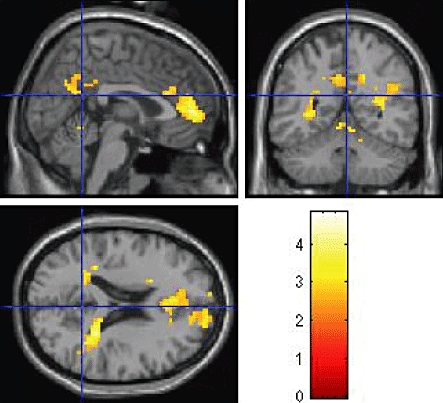
Group interaction: Areas highlighted for higher activation in Fagerström Test for Nicotine Dependence (FTND)-high group versus the pooled sample of FTND-low group, problem gamblers (PRG) and healthy controls (HC) at coordinates 3, −51, 21. No significant relations between Beck Depression Inventory-II, or Conners Adult ADHD Rating Scales scores and blood oxygenation level-dependent response when viewing smoking-related pictures versus neutral pictures were present in the FTND-high group, FTND-low group, PRG or HC

No significant group by condition interactions were observed for smoking pictures in HSM compared with PRG or HC. Greater activation was present in the ventromedial prefrontal cortex (VMPFC) bilaterally, in the rostral ACC bilaterally and in the left VLPFC in the FTND-high group compared with HC and in the FTND-high group compared with the FTND-low group. Similar effects were observed when comparing the FTND-high group with PRG (see Table 3 and Fig. 4). In addition, in the FTND-high group, activation in the left precuneus, right insula and left middle and superior temporal gyri was greater than in the FTND-low group. No significant group by condition interactions were observed in the FTND-low group compared with either HC or PRG.

**Table 3:** Cue-reactivity task: BOLD activations for group interactions: smoking-related pictures versus neutral pictures.

	*FTND-high > Healthy Controls*					*FTND-high > FTND-low*					*FTND-high > Problem Gamblers*				
		*MNI coordinates*					*MNI coordinates*					*MNI coordinates*			
*Nicotine vs. Neutral Pictures*	*L/R*	*x*	*y*	*z*	*Z value*	*L/R*	*x*	*y*	*z*	*Z value*	*L/R*	*x*	*y*	*z*	*Z value*
Prefrontal															
Ventromedial	L	−12	54	−3	3.61	L	−12	51	−3	3.51	L	−6	66	15	3.46
	R	12	51	0	3.71	R	9	51	6	3.95	R	9	60	15	3.30
Ventrolateral	L	−33	51	6	3.83						R	15	51	3	3.65
Superior frontal gyrus						R	33	42	42	3.39					
rostral ACC	L	−6	42	9	3.79	L	−6	42	9	3.88	L	−6	48	3	4.07
		−3	48	6	3.54	R	9	42	6	3.56	R	6	45	6	3.68
	R	6	42	9	3.55										
Parietal	L	−9	−60	36	3.61						L	−3	−48	33	3.65
Middle temporal gyrus						L	−51	6	−21	4.27					
Superior temporal gyrus						L	−45	9	−15	3.52					
Insula						R	36	9	−9	3.44					
Precuneus						L	−6	−54	36	3.56					

#### Correlations between BOLD activation, subjective craving, BDI-II and CAARS

Regression analyses indicated a positive relation between subjective craving for gambling after scanning in PRG and BOLD activation in the VLPFC, left anterior insula and left caudate head when viewing gambling pictures versus neutral pictures (see Table 2). A positive relation between subjective craving for nicotine after scanning in HSM and BOLD activation in the VLPFC and left amygdala region during viewing of smoking-related pictures versus neutral pictures was present (Table 4).

**Table 4:** Cue Reactivity Task: correlations between BOLD activations and self-reported craving levels in problem gamblers and heavy smokers

	*Regression analyses*									
	*Gambling versus neutral pictures with gambling urge in problem gamblers*					*Smoking-related versus neutral pictures with smoking urge in heavy smokers*				
*Positive correlations*	*L/R*	*x*	*y*	*z*	*Z value*	*L/R*	*x*	*y*	*z*	*Z value*
VLPFC	L	−39	45	−3	4.32	L	−36	6	60	3.56
dACC	L	−6	27	39	3.31					
Anterior insula	L	−48	21	−3	4.02					
Amygdala region						L	−18	3	−21	3.51
Caudate head	L	−15	15	6	3.29					

Group interactions are reported at *P* < 0.001 unless indicated otherwise. BOLD = blood oxygenation level-dependent; dACC = dorsal anterior cingulate cortex; VLPFC = Ventrolateral prefrontal cortex.

No significant relations between BDI-II or CAARS scores and regional cerebral blood flow changes during viewing gambling or smoking-related pictures versus neutral pictures were present in PRG, HSM or HC.

## DISCUSSION

This is the first study investigating cue reactivity to gambling stimuli in treatment-seeking PRG compared with HSM and HC, using an fMRI event-related picture paradigm. PRG showed higher brain activation compared with HC and HSM when viewing gambling pictures (compared with neutral pictures) in brain areas related to visual information processing and memory (bilateral occipital cortex, parahippocampal gyrus), and emotion and motivation (amygdala region, VLPFC). Specifically, upregulation of visual information processing areas has been related to altered dopaminergic transmission in neural systems implicated in substance dependence: (1) an emotion/motivational and memory/learning circuit, including orbitofrontal, subcallosal cortex, amygdala and hippocampus; and (2) an attention/control circuit, including dorsal prefrontal and ACC (Breiter & Rosen 1999; Goldstein & Volkow 2002; Kalivas & Volkow 2005). Higher activation in PG in these visual information processing areas may thus be related to a higher saliency of gambling stimuli, through innervations of dopamine pathways from the nucleus accumbens, ventral tegmental area and limbic areas to this visual system. Similar brain areas were found to be activated in fMRI cue reactivity studies of smokers and alcohol-dependent persons (George *et al*. 2001; Due *et al*. 2002; Myrick *et al*. 2004). Higher activation of the amygdala region and parahippocampal gyrus indicates that gambling pictures activated the emotion/motivation and memory-related circuitry more in PRG than in HSM and HC. The parahippocampal gyrus is involved in processing complex visual information, receives input from the nucleus accumbens and amygdala, and is an important afferent pathway to the hippocampus. Cue reactivity studies of problem gambling, alcohol dependence and nicotine dependence have also reported brain activation in the parahippocampal gyrus (Crockford *et al*. 2005; Smolka *et al*. 2006; Park *et al*. 2007). This study is the first to show involvement of the amygdala region in a cue-reactivity study in PRG, and to observe that activation in brain areas such as insular cortex and caudate nucleus is associated with self-reported gambling craving. These findings point to the persistent emotional relevance of gambling stimuli in patients currently in treatment for gambling problems.

All PRG were being treated for PG when they participated in the study, and reported an average duration of gambling problems of 13 years (data not shown). The two fMRI cue reactivity studies in PG present in the literature (Potenza *et al*. 2003; Crockford *et al*. 2005) focused on community-recruited PRG, and did not report amygdala, insular cortex or caudate nucleus activation. The findings of this study suggest that cue reactivity in chronic PRGs seeking treatment may be related more strongly to brain reactivity in emotional and motivational circuitries than cue reactivity in (non-chronic) PRGs who are not in treatment.

Differences in brain activation patterns to smoking pictures between the FTND-high smokers and HC or PRG were most consistently present in VLPFC, VMPFC and rostral ACC, conforming with previous fMRI cue reactivity studies in smokers (David *et al*. 2005; Lee *et al*. 2005; McClernon *et al*. 2005, 2008). The lack of an effect of cue-reactivity in the FTND-low HSM group compared with the PRG or the HC group is likely related to the lower level of nicotine dependence in this subgroup. It has been reported that FTND scores positively correlate with regional brain reactivity to smoking cues (Smolka *et al*. 2006; McClernon *et al*. 2008). Therefore, in future studies, the selection of a more homogeneous group of smokers, with a minimum score on the FTND or a formal DSM-IV ND diagnosis would be advisable.

In addition to our findings of higher brain activation in VMPFC and rostral ACC in the FTND-high smokers compared with the other groups, we observed that smoking urge in HSM correlated positively with activity in brain areas related to emotion and reward/motivational processing (amygdala and VLPFC), areas previously implicated in craving for smoking (David *et al*. 2005; McClernon *et al*. 2008).

### Limitations

Although we observed increased brain activation in response to gambling pictures in PRG and to smoking cues in the FTND-high HSM group, viewing these pictures elicited only a trend for higher self-reported craving in PRG, whereas in HSM no effects of the cue reactivity task on smoking urges were present. Changes in subjective craving before and after the task may have been limited in our study because of the timing of the measurement: a paper and pencil craving questionnaire was filled out after leaving the scanner, when immediate effects of the task on craving may have subsided. In future research, computerized craving measures administered in the scanner, halfway or immediately after the cue reactivity task, are therefore preferable.

After recruiting the HSM group, it became clear that FTND scores differed substantially within this group. Therefore, *post hoc* comparisons were made between two subgroups of HSM: an FTND-high group and an FTND-low group. The differential findings in the FTND-high and FTND-low groups imply that it is important to include a measure of nicotine dependence severity in cue reactivity studies in smokers, in addition to selecting smokers based on the number of cigarettes they smoke. The group sizes of the FTND subgroups were small (*n* = 10 and *n* = 8, respectively), and therefore the results regarding these subgroups have to be interpreted with caution. Studies in larger groups of smokers differing in FTND scores should be done to replicate these preliminary findings.

### Conclusion

This study demonstrates that viewing gambling pictures (as opposed to neutral pictures) is related to greater brain activation in visual processing, emotion-motivation and attentional-control brain circuitry in treatment-seeking PRG, compared with HC and HSM, and that this activation is positively related to gambling urges. These effects are consistent with those observed in substance-dependent persons (George *et al*. 2001; Myrick *et al*. 2004; Franklin *et al*. 2007). In the present study, we did observe increased brain reactivity to smoking cues in persons with FTND scores indicating moderate nicotine dependence compared with HC, but did not find differences in persons with an FTND score indicating low nicotine dependence. Higher smoking urge in HSM was associated with increased activity in reward and emotion-related brain areas. Future research needs to establish whether the long-term effects of gambling cues on brain activation in PRG in treatment are related to relapse in problem gambling.
